# Comparing Two Types of Rabbit ATG prior to Reduced Intensity Conditioning Allogeneic Hematopoietic SCT for Hematologic Malignancies

**DOI:** 10.1155/2015/980924

**Published:** 2015-03-22

**Authors:** Sandra Paiano, Eddy Roosnek, Yordanka Tirefort, Monika Nagy-Hulliger, Stavroula Masouridi, Emmanuel Levrat, Michael Bernimoulin, Saadia Huguet, Alessandro Casini, Thomas Matthes, Kaveh Samii, Jakob R. Passweg, Yves Chalandon

**Affiliations:** Division of Hematology, Geneva University Hospital and University of Geneva, Geneva, Switzerland

## Abstract

Different rabbit polyclonal antilymphocyte globulins (ATGs) are used in allogeneic hematopoietic stem cell transplantation (alloHSCT) to prevent graft-versus-host disease (GvHD). We compared 2 different ATGs in alloHSCT after reduced intensity conditioning (RIC) for hematological malignancies. We reviewed 30 alloHSCT for hematologic malignancies performed between 2007 and 2010 with fludarabine and i.v. busulfan as conditioning regimen. Patients alternatingly received Thymoglobulin or ATG-F. Median followup was 3.3 (2.5–4.5) years. Adverse events appeared to occur more frequently during Thymoglobulin infusion than during ATG-F infusion but without statistical significance (*P* = 0.14). There were also no differences in 3-year overall survival (OS), disease-free survival (DFS), relapse incidence, and transplant related mortality (TRM) in the Thymoglobulin versus ATG-F group: 45.7% versus 46.7%, 40% versus 33.7%, 40% versus 33.3%, and 20% versus 33.3%. The same held for graft failure, rejection, infectious complications, immune reconstitution, and acute or chronic GvHD. In patients transplanted for hematologic malignancies after RIC, the use of Thymoglobulin is comparable to that of ATG-F in all the aspects evaluated in the study. However due to the small number of patients in each group we cannot exclude a possible difference that may exist.

## 1. Introduction

Antithymocyte globulins (ATGs) are used as immunomodulatory agents for prevention and treatment of graft-versus-host disease (GvHD) [1] in allogeneic hematopoietic stem cell transplantation (alloHSCT), for prevention and treatment of solid organ graft rejection, for treatment of aplastic anemia, and occasionally for treatment of other autoimmune disorders [2, 3]. These polyclonal immunoglobulins are IgG preparations from rabbits immunized with human thymocytes (Thymoglobulin) or with the T-acute lymphoblastic leukemia cell line Jurkat (ATG-Fresenius, ATG-F) [4]. ATG depletes T lymphocytes by induction of apoptosis or complement-dependent lysis. Furthermore, it may add immune suppression by modulation of surface molecules mediating leukocyte/endothelium interactions, induction of B-cell apoptosis, interference with dendritic cells properties, induction of regulatory T cells, or induction of NK T cells [5].

Differences between safety and efficacy of different brands of ATG are not well understood and only few studies have addressed these questions [6–9].

The aim of this analysis was to compare the impact of these two rabbit polyclonal antilymphocyte globulins on outcome in alloHSCT after reduced intensity conditioning regimen (RIC) for hematological malignancies.

## 2. Patients and Methods

Our report is a retrospective study of 30 consecutive patients transplanted between 2007 and 2010 after RIC consisting of ATG in combination with fludarabine 30 mg/m^2^/day for 5 days and busulfan i.v. 3.2 mg/kg/day for 2 days or fludarabine 30 mg/m^2^/day for 5 days and melphalan 70 mg/m^2^/day for 2 days (Hodgkin disease patients only). In order to avoid cost containment measures, we alternated between treatment with Thymoglobulin 2.5 mg/kg/day from day 5 to day 3 before transplant and ATG-F 5 mg/kg/day from day 6 to day 2. Twelve patients were transplanted for AML, the others for undifferentiated acute leukemia [1], lymphoma [9], multiple myeloma [2], chronic myelomonocytic leukemia [3], myelodysplastic syndrome [2], or myelofibrosis [1]. Stem cells came from peripheral blood mononuclear cells for all patients except for one patient in the ATG-F group who received bone marrow. Immunosuppression consisted of cyclosporine 4 mg/kg/day in continued infusion, mycophenolate mofetil for 1 month, and methylprednisolone 1000 mg during the 2 days before transplantation. Sixteen patients received a graft T cell depleted with 20 mg alemtuzumab* in vitro* followed the next day by an infusion of 100 × 10^6^ CD3/kg for related donors and 0.35 × 10^6^ CD3/kg for unrelated donors [10]. Median followup was 3.3 (2.5–4.5) years with no significant difference between groups receiving different ATG. We reviewed patient age, gender, diagnosis, remission status at transplant, CMV serostatus, reason for RIC instead of standard conditioning, type of donor, use of T cell depletion with Campath (alemtuzumab), T lymphocyte add-back dose, blood group compatibility, and GvHD prophylaxis and found no differences between the two groups (Table 1).

### 2.1. Outcomes

Outcomes analyzed were toxicity (incidence of adverse events during infusion of ATG), graft failure, rejection, infectious complications (infectious complications leading to hospitalization, intravenous antibiotherapy, or antiviral treatment (excluding preemptive treatment for CMV reactivation without clinical infection)), effects on immune reconstitution, acute and chronic GvHD, treatment related mortality (TRM), relapse incidence, and overall survival. Immune reconstitution was assessed by CD3, CD4, and CD8 counts and immunoglobulin levels (IgG, IgA, and IgM).

### 2.2. Statistical Analysis

Appropriate parametric or nonparametric tests were used to compare groups for continuous or categorical variables. Overall survival (OS) and disease-free survival (DFS) were estimated using the Kaplan-Meyer method [11]; the log-rank test was used for comparison. Cumulative incidence was used for relapse, TRM, and GvHD. Relapse was used as competing risk for TRM incidence and also for GvHD incidence.

## 3. Results

### 3.1. Toxicity

There was a tendency to a higher incidence of adverse events during the Thymoglobulin perfusion compared to ATG-F, *P* = 0.14 (Table 2). Chills and/or fever occurred in 7 patients (2 patients had fever, 2 had chills, 2 had fever and chills including 1 with arterial hypotension, and 1 had fever with osteoarticular pain) and acute hepatic cytolysis in one patient in the Thymoglobulin group. In the ATG-F group, osteoarticular pain occurred during 2 infusions, fever and chills during one, and chills during another infusion.

### 3.2. Graft Take/Rejection

Time to engraftment was similar in both groups, with neutrophils reaching >0.5 G/L at a median time of 17 days (7–21) in the ATG-F group and 17.5 days (11–22) in the Thymoglobulin group (*P* = 0.60). Neutrophils reached levels above 1.5 G/L at a median time of 19 days (10–22) in the ATG-F group and 18 days (12–24) in the Thymoglobulin group (*P* = 0.76). Thrombocytes reached levels above 20 G/L at a median of 8.5 days (0–25) in the ATG-F group and 10 days (0–85) in the Thymoglobulin group (*P* = 0.46) and were at 50 G/L at a median time of 13 days (0–35) in the ATG-F group and 12 days (0–40) in the Thymoglobulin group (*P* = 0.52). There was one graft failure in each group (Table 2). Two rejections occurred in the ATG-F group and 3 in the Thymoglobulin group with similar median times to rejection (ATG-F group, 48 days, range 39–57; Thymoglobulin, 39 days, range 28–72, *P* = 0.93).

### 3.3. Infections

The number of patients suffering from severe infections after the period of aplasia in the two groups was similar (9 in the ATG-F group and 7 in the Thymoglobulin group, *P* = 0.46) (Table 2). They consisted mainly of respiratory tract infections and bacteremia and some patients suffered from more than one. CMV viremia reactivation occurred in 11 patients in the ATG-F group and in 10 in the Thymoglobulin group, *P* = 0.69 (Table 2). Owing to the preemptive treatment with ganciclovir or valganciclovir, no clinical infection occurred.

### 3.4. Immune Reconstitution

We measured immune reconstitution by the number of CD4- and CD8-positive T cells and by dosage of serum immunoglobulins. At 1 year after transplantation, the number of CD4-positive T cells reached >200/uL in 3 patients treated with ATG-F and in 5 treated with Thymoglobulin (*P* = 1.00), >400/uL in 0 and 2 patients in the respective groups (*P* = 0.18). CD8 counts >300/uL were reached in 6 and 5 patients, respectively (*P* = 1.00). Eight patients in the ATG-F group and 7 patients in the Thymoglobulin group had an IgG level >5 g/L (*P* = 1.00) while the level of IgG, IgA, and IgM was normal in 5 and 4 patients, respectively (*P* = 1.00).

### 3.5. GvHD

**Figure 1:**
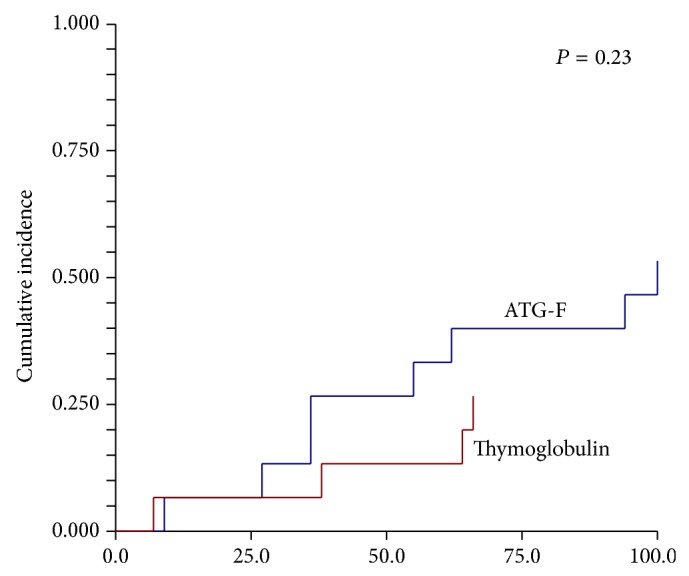
Cumulative incidence of aGvHD grades I–IV at day 100, split by different ATG, 53.3% (95% CI 33.2–85.6) in the ATG-F group, 26.7% (95% CI 11.5–61.7) in the Thymoglobulin group, *P* = 0.23.

The cumulative incidence of acute GvHD (aGvHD)* grades I*–*IV* at day 100 was twice as high (53.3%, 95% CI 33.2–85.6%) in the ATG-F group as in the Thymoglobulin group (26.7%, 95% CI 11.5–61.7%) but this difference did not reach statistical significance, *P* = 0.23 (Table 2, Figure 1). Two GvHD grade I and 6 GvHD grade II occurred in the ATG-F group and 2 GvHD grade I, 1 grade II, 1 GvHD grade III, and 1 grade IV in the Thymoglobulin group. The cumulative incidence of mainly extensive chronic GvHD at 3 years was 13.3% (95% CI 3.7–48.5%) in the ATG-F group and 20.0% (95% CI 7.3–55.0%) in the Thymoglobulin group, *P* = 0.87 (Table 2).

### 3.6. OS, DFS, TRM, and Relapse

**Figure 2:**
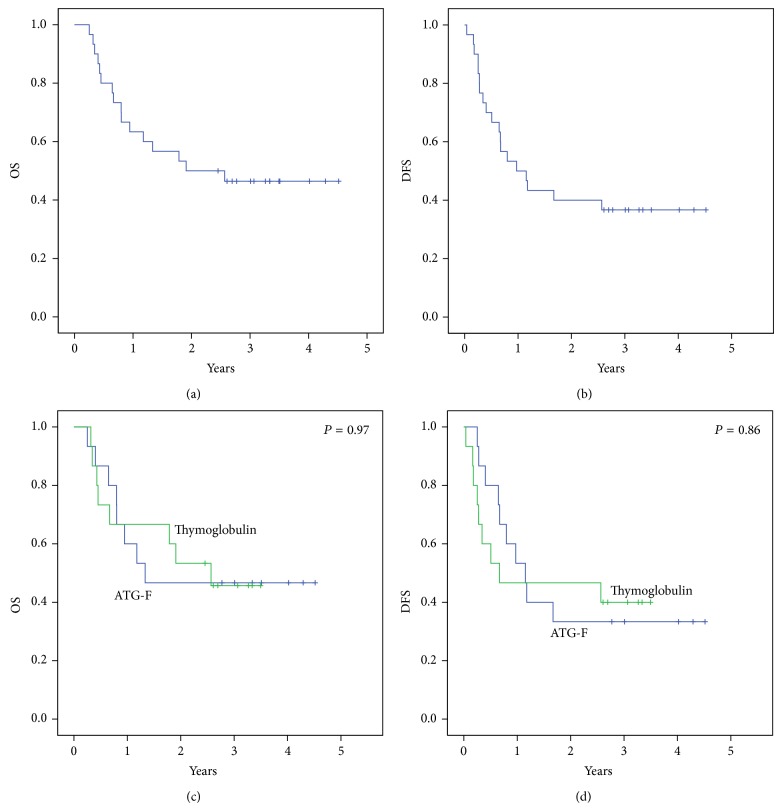
Three years (a) OS (46.4% (95% CI 28.4–64.4%)), (b) DFS (36.7% (95% CI 19.1–54.3%)), (c) OS split by different ATG (45.7 (95% CI 19.7–71.7%) in the Thymoglobulin group and 46.7 (95% CI 20.7–72.7%) in the ATG-F group, *P* = 0.97), and (d) DFS split by different ATG (40.0 (95% CI 14.8–65.2%) in the Thymoglobulin group and 33.7 (95% CI 8.9–58.1%) in the ATG-F group, *P* = 0.86).

OS and DFS at 3 years were 46.4% (95% CI 28.4–64.4%) and 36.7% (95% CI 19.1–54.3%), respectively, (Figures 2(a)-2(b)), without statistical differences between both groups. At three years OS was 45.7 (95% CI 19.7–71.7%) and 46.7 (95% CI 20.7–72.7%), *P* = 0.97. At 3 years DFS was 40.0 (95% CI 14.8–65.2%) and 33.7 (95% CI 8.9–58.1%), *P* = 0.86, in the Thymoglobulin and ATG-F groups, respectively, (Figures 2(c)-2(d)).

We observed tendencies to a lower TRM and more relapses in the Thymoglobulin group but this did not reach statistical significance. Cumulative incidence of TRM at 3 years was 33.3%, 95% CI 16.3–68.2% in the ATG-F group, and 20%, 95% CI 7.3–55% in the Thymoglobulin group, *P* = 0.67 (Table 2). Cause of death was infection (3 in each group), relapse (3 in the ATG-F group and 4 in the Thymoglobulin group), GvHD (1 in each group), or hemorrhage (1 in the ATG-F group). The cumulative incidence of relapse at three years (ATG-F group 33.3%, 95% CI 16.3–68.2%, and Thymoglobulin group 40%, 95% CI 21.5–74.3%) was not statistically different, *P* = 0.55 (Table 2).

## 4. Discussion

We have analyzed the outcome of two different rabbit polyclonal antilymphocyte globulins in alloHSCT after RIC for hematological malignancies.

In our small cohort of 30 patients, we did not find any statistically significant differences between the two groups regarding all parameters tested (i.e., toxicity, engraftment, infection rate, immune reconstitution, GvHD incidence, TRM, or DFS). Given the small groups analyzed, it is not possible to see if there are differences, even if there were some. However, tendency towards more relapse, less TRM, and less acute GvHD was noticed in the Thymoglobulin group. This may point to a more T cell depleting potential in the doses used.

To our knowledge, our study is the first to compare these two ATGs in alloHSCT for patients suffering from hematological malignancies. A single center retrospective Japanese study [6] comprising 3 patients receiving Thymoglobulin (2.5 mg/kg/day from day 5 to day 2 before transplant) and 4 receiving ATG-F (5 mg/kg/day from day 7 to day 3 before transplant) has compared these ATGs in alloHSCT for aplastic anemia. They noticed no acute GvHD grade ≥II, nor rejection, but they observed CMV reactivation in 3/3 patients who had received Thymoglobulin and in 2/4 of the ATG-F group. CD4 and CD8 lymphocytes recovered later in the Thymoglobulin group than in the ATG-F group (data based on values on day 60). These results suggested that Thymoglobulin had a stronger immunosuppressive effect than ATG-F. In our study, we confirmed these high rates of CMV reactivation (10/15 in the Thymoglobulin and 11/15 in the ATG-F group) but we did not see a difference in CD4 and CD8 lymphocyte recovery at one year.

ATG is used to prevent rejection after solid organ transplantation. Also here, some studies have reported a possible more immunosuppressive effect of Thymoglobulin. In a single center retrospective French study published in 2004, comprising 194 renal transplanted patients [7], 65 of whom received Thymoglobulin (2–5 mg/kg day 0 and 1-2 mg/kg days 1 to 4 after transplant) and 129 ATG-F (9 mg/kg day 0 and 3 mg/kg days 1 to 4 after graft), more CMV reactivation occurred in the Thymoglobulin than in the ATG-F group (37% versus 23%, *P* = 0.02). Furthermore, in the Thymoglobulin group more patients developed posttransplant malignancies (12.3% versus 3.9%, *P* = 0.01) and/or died (13.8 versus 3.9%; *P* = 0.005). An Italian single center prospective randomized trial in heart transplantation [8] with 20 patients in the Thymoglobulin group (2.5 mg/kg/day for 5 days after transplant) and 20 in ATG-F group (2.5 mg/kg/day for 7 days after transplant) found no difference in rejection and survival, but more CMV reactivations in the Thymoglobulin group (65% versus 30%; *P* = 0.002). Furthermore new CMV infections occurred only in the Thymoglobulin group (20%, *P* = 0.05). No secondary malignancies were observed (followup of 32.8 ± 8.9 months). By contrast, a Swiss single center prospective randomized study from 2002 [9] comparing Thymoglobulin 2.5 mg/kg days 1 to 5 after transplant (*n* = 26) and ATG-F 3–3.5 mg/kg days 1 to 5 after transplant (*n* = 24) in the induction treatment for heart transplantation found no difference in survival, acute rejection, or infection rate for patients followed one year.

A more immunosuppressive effect of Thymoglobulin may be owed to a more efficient depletion of T cells. Twenty years ago, different preparations of horse and rabbit ATG have been compared [12], including Thymoglobulin and ATG-F, and the latter appeared to bind somewhat less efficiently to many of the surface antigens on T cells. However, another study showed no difference in T cell cytotoxicity of Thymoglobulin and ATG-F [13]. Hence, differences will be relatively small, also because the effective doses of both ATGs have been titrated to calibrate the risk of infection and taking the benefit against GvHD and graft failure [14–16].

## 5. Conclusions

In conclusion, subject to the small groups analyzed, our study does not show any statistically significant differences between Thymoglobulin and ATG-F regarding all the parameters tested. It may therefore be worthwhile to compare more in depth these two antilymphocyte globulins, in a larger study, to ascertain whether the tendency to some difference shown in the literature is real or not. However due to the small number of patients in each group we cannot exclude a possible difference that may exist. In fact, if there are some, the choice of the product should be based on the specific properties of each one and so adapted to the patient risk status.

## Figures and Tables

**Table 1:** Patients, conditioning, and graft characteristics.

Patients, conditioning, and graft characteristics	ATG-F	Thymoglobulin	*P*
	*n* = 15	*n* = 15	
Age at transplant, median (range, years)	59 (40–69)	51 (22–68)	0.49
Number of men, *n* (%)	8 (53)	9 (60)	0.72
Group diagnosis, *n* (%)			
AML	6 (40)	6 (40)	1.00
MDS	2 (13)	0	0.46
CMML	0	3 (20)	0.23
MF	1 (7)	0	1.00
NHL/HD	5 (33)	4 (27)	0.69
MM	1 (7)	1 (7)	1.00
Undifferentiated AL	0	1 (7)	1.00
Complete remission at transplant, *n* (%)	15 (100)	13 (87)	0.48
CMV serostatus donor/recipient, *n* (%)			
Positive recipient	11 (73)	12 (80)	0.67
Positive donor	7 (47)	8 (53)	0.72
Reason for RIC, *n* (%)			
Prior autologous SCT	8 (53)	8 (53)	1.00
Age	4 (27)	4 (27)	1.00
Other	3 (20)	3 (20)	1.00
Chemotherapy agents, *n* (%)			
Fludarabine-busulfan	13 (87)	12 (80)	0.67
Fludarabine-melphalan	2 (13)	3 (20)	1.00
Related donor, *n* (%)	4 (27)	5 (33)	0.69
HLA-identical	4 (27)	5 (33)	0.69
Unrelated donor, *n* (%)	11 (73)	10 (67)	0.69
0 mismatch	8 (53)	6 (40)	0.46
≥1 mismatch	3 (20)	4 (27)	0.67
Men donor, *n* (%)	9 (60)	13 (87)	0.10
Sex mismatch in GvHD sense, *n* (%)	4 (27)	1 (7)	0.33
Partial T cell depletion, *n* (%)	7 (47)	9 (60)	0.46
T cell dose, median (range, 10^*E*^6 CD3/kg)	5 (0.35–130)	5 (0.35–100)	0.78
Blood group incompatibility, *n* (%)			
Major	4 (27)	4 (27)	1.00
Minor	4 (27)	5 (33)	0.91
Posttransplant immunosuppression, *n* (%)	15 (100)	15 (100)	1.00

**Table 2:** Complications.

Complications	ATG-F	Thymoglobulin	*P*
	*n* = 15	*n* = 15	
Adverse effect of antilymphocyte globulin perfusion, *n* (%)	4 (27)	8 (53)	0.14

Graft failure, *n* (%)	1 (7)	1 (7)	1.00

Rejection, *n* (%)	2 (13)	3 (20)	1.00

Relapse, *n* (%)	5 (33)	7 (47)	0.46

Cumulative incidence of relapse at 3 years (%)	33.3 (16.3–68.2)	40 (21.5–74.3)	0.55

CMV reactivation, *n* (%)	11 (73)	10 (67)	0.69

Severe infection after aplasia, *n* patients (%)	9 (60)	7 (47)	0.46

Respiratory infections, *n*	2 RSV 1 *Staph. aureus* pneumonia	2 H1N1 influenza 2 bacterial pneumonia 1 invasive lung aspergillosis	

Bacteremia, *n*	2 *Staphylococcus* 1 *Kytococcus schroeteri *	1 multiple (*E. coli*, *Pseudomonas sp.*, *Listeria monocytogenes*)	

Other infections, *n*	3 BK-virus cystitis 1 Microsporidiosis 3 *E. coli* urinary 1 HHV-6 encephalitis 2 genital HSV-1	1 gastroenteritis 1 acute middle otitis 1 generalized aspergillosis 1 large spectrum beta-lactamase enterobacteria urinary	

Acute GvHD grade I, *n* (%)	2 (13)	2 (13)	1.00

Acute GvHD grades II–IV, *n* (%)	6 (40)	3 (20)	0.23

Cumulative incidence of aGvHD grades I–IV at day 100 (%)	53.3 (33.2–85.6)	26.7 (11.5–61.7)	0.23

Chronic limited GvHD, *n* (%)	0	1 (7)	1.00

Chronic extensive GvHD, *n* (%)	4 (27)	3 (20)	0.67

Cumulative incidence of cGvHD at 3 years (%)	13.3 (3.7–48.5)	20.0 (7.3–55.0)	0.87

Death, *n* (%)	8 (53)	8 (53)	1.00

TRM, cumulative incidence at 3 years (%)	33.3 (16.3–68.2)	20 (7.3–55)	0.67

OS, cumulative incidence at 3 years (%)	46.7 (20.7–72.7)	45.7 (19.7–71.7)	0.97

DFS, cumulative incidence at 3 years (%)	33.7 (8.9–58.1)	40.0 (14.8–65.2)	0.86
